# Adsorption Characteristics of Anionic Surfactant Sodium Dodecylbenzene Sulfonate on the Surface of Montmorillonite Minerals

**DOI:** 10.3389/fchem.2018.00390

**Published:** 2018-10-02

**Authors:** Xiaoming Ni, Zhiheng Li, Yanbin Wang

**Affiliations:** ^1^School of Energy Science and Engineering, Henan Polytechnic University, Jiaozuo, China; ^2^Collaborative Innovation Center of Coalbed Methane and Shale Gas for Central Plains Economic Region Henan Province, Jiaozuo, China; ^3^College of Geo-science and Surveying Engineering, China University of Mining and Technology, Beijing, China

**Keywords:** montmorillonite, sodium dodecylbenzene sulfonate, adsorption kinetics, surface adsorption, interlayer adsorption

## Abstract

The adsorption characteristics of sodium dodecylbenzene sulfonate (SDBS) on the surface of montmorillonite can lay a foundation for obtaining the optimum concentration of the anionic surfactant. The best absorption wavelength of SDBS was determined using an ultraviolet spectrophotometer. The standard curves of concentration and absorbance of SDBS were established. The amount of SDBS adsorbed on the surface of montmorillonite at various concentrations was calculated by stirring adsorption method. Scanning electron microscopy–energy dispersive X-ray spectrometry (SEM-EDS), X-ray diffraction (XRD), zeta potentiometer, and Fourier transform infrared (FTIR) spectroscopy were used to observe the changes of the structure, main ions, interlayer spacing, potential, and main functional groups on the montmorillonite surface before, and after, adsorption. The test results of SEM with EDS (SEM–EDS) showed that the surface of the montmorillonite after SDBS adsorption was rougher, and the adsorption capacity of the surface was enhanced as the SDBS concentration increased. The XRD results indicated that SDBS adsorbed on the interlayer of montmorillonite repulsed interlayer water and reduced the interlayer water content. With the increase of SDBS concentration, the interlayer spacing of the montmorillonite available for adsorbing SDBS decreased further. Additionally, interlayer adsorption and surface adsorption exist simultaneously in montmorillonite in SDBS solution. The distribution of total adsorption capacity of SDBS in the layers and on the surface of montmorillonite accords with the adsorption result simulated by a pseudo-second-order kinetic model. The increase in concentration of SDBS adsorbed by montmorillonite is the main reason for the decreased initial adsorption rate. The zeta potential test showed that the addition of H^+^ to the SDBS solution could reduce electrostatic repulsion and promote the adsorption of SDBS on montmorillonite. The results of this study provide an experimental basis for the study of the mechanism of SDBS adsorption on montmorillonite.

## Introduction

Montmorillonite, as a clay mineral carrying negative charges on the surface, is used as an adsorbent for most organic compounds (Prost and Yaron, 2001; Bhattacharyya and Gupta, 2008; Xi et al., 2010; Zhu et al., 2014). Owing to a lot of clay minerals being contained in shale, surfactant in the fracturing fluid can be adsorbed by montmorillonite in the clay layers in reservoir reconstruction, which influences the performance of the fracturing fluid. Therefore, analysis of the adsorption characteristics of anionic surfactants on the surface of montmorillonite is of reference significance to the optimum selection of fracturing fluid composition.

Finding out the changes in adsorption capacity and adsorption kinetic characteristics of a surfactant on the surface of clay minerals under different time, temperature, and concentration conditions can lay a foundation for studying adsorption mechanisms. The test methods for studying adsorption capacity of surfactant on the surface of clay minerals mainly include nuclear magnetic resonance (NMR), spectroscopic ellipsometry, and the static adsorption method combined with the use of an ultraviolet–visible spectrophotometer or a fluorophotometer. The NMR determination method is mainly used to test the T1 and T2 spectra of the samples before, and after clay minerals adsorb surfactant, so as to calculate their surface adsorption capacity. The accurate explanations of the spectra can determine the accuracy of test results to a great extent (Söderlind and Stilbs, 1991; Totland et al., 2011). Spectroscopic ellipsometry can be employed to calculate the thickness of the adsorbed layers on the surface of the samples by testing the differences between polarization states of the incident and reflected beams for the samples before and after adsorption, and then deducing the amount of adsorption by integrating other relevant parameters: however, the large test errors in the method for nonuniform surfaces result in inaccurate results (Luciani and Denoyel, 1997; Denoyel, 2002). The static adsorption method combining with test instruments, such as an ultraviolet–visible spectrophotometer, a fluorophotometer, a total organic carbon analyzer, or a liquid chromatography analyzer, is used to test the adsorption capacity of a surfactant on the surface of clay minerals under different conditions (Liu et al., 2016). The method is mainly used to test the changes in concentration of surfactant solutions before, and after, adsorption, so as to obtain their adsorption capacity with reference to the corresponding standard curve. Compared with other methods, this method is easy to operate, widely used, exploits mature technologies, and confers the advantages of timely measurement. This study used the method to test the adsorption capacity of the surfactant on the surface of montmorillonite.

By testing the adsorption capacity of a surfactant on the surface of clay minerals over different times, the changes in the adsorption capacity with time were obtained. By using different adsorption kinetics equations for fitting, the adsorption kinetics models with a high correlation were determined. At present, the pseudo-first-order kinetic model and pseudo-second-order kinetic model are widely utilized in research in the adsorption of surfactants on clay minerals. In general, it is considered that the pseudo-first-order kinetic model mainly shows physical adsorption, while adsorption meeting the pseudo-second-order kinetic model is mainly chemical adsorption (Lagergren, 1898; Ho and Mckay, 1999; Nandi et al., 2009; Simonin, 2016). This research obtained the main adsorption types of anionic surfactants on the surface of montmorillonite by fitting the two kinetics models and combining these with an assessment of the infrared spectra.

The surfactant may be adsorbed on the surface or the interlayer of clay minerals. The following methods are mainly used for researching adsorption of the surfactant on the surface of clay minerals: scanning electron microscopy (SEM) observations, energy dispersive X-ray spectrometry (EDS) analysis, and X-ray photoelectron spectroscopy (XPS) analysis (Moraru, 2001; Zhou et al., 2009; Li and Wu, 2010; Park et al., 2011; Liu et al., 2016). The SEM observations can reveal the morphological changes of the surface of clay minerals before, and after, adsorption. The EDS and XPS analyses are mainly used to analyze the element types present and their relative contents according to the corresponding wavelengths of X-rays of different elements on the surface of clay minerals. The XRD test can reveal interlayer information of clay minerals based on characteristic peaks and angles in spectra, and is widely applied in the study of interlayer changes of clay minerals. This study investigated the adsorption of anionic surfactants on the surface of clay minerals by combining SEM observations with EDS analysis. Furthermore, by utilizing an XRD instrument, the change characteristics of the interlayer of clay minerals before, and after, adsorption were investigated.

Electrostatic force is found to influence the adsorption performance of surfactants on the surface of clay minerals. To reveal the effects of pH value on the adsorption capacity, this study tested the zeta potential of clay minerals in solutions with different pH values by using a zeta potential analyzer (Kaya and Yukselen, 2005; Navrátilová and Maršálek, 2012; Yu et al., 2018). Based on this, the influences of pH value on the adsorption of clay minerals were obtained. This research attempts to provide an experimental basis for investigating the adsorption mechanisms of anionic surfactants on the surface of montmorillonite.

## Experimental methods

### Experimental instruments and samples

The SDBS used in the experiment was produced by the Fangzheng Reagent Factory, Beichen District, Tianjin, China. Montmorillonite minerals were provided by Huashou Mineral Products Company, Lingshou County, Hebei Province, China. The experimental instruments included an HH-ZK1 thermostat water bath, a magnetic stirrer, and an Evolution 201 ultraviolet spectrophotometer (Thermo Fisher Scientific Inc., USA). The test wavelength of the ultraviolet spectrophotometer ranged from 190 to 1,100 nm, and the wavelength accuracy was ± 0.8 nm. Moreover, it was able to test the absorbance in the range of −0.3 to 4.0 A.

The SEM–EDS with an energy spectrometer was from Carl Zeiss AG, Germany. The resolution of the SEM was 3.0 nm in high-vacuum mode and 4.0 nm in low-vacuum mode. The magnification varied from 5 to 1 × 10^6^ and the EDS energy resolution changed from 0 to 132 eV. It was able to analyze elements from Be to U.

The D8 X-ray diffractometer was manufactured by BRUKER-AXS, Germany. The divergent slit, antiscatter slit, Sola slit, and receiving slit were 1.0 mm, 1.0 mm, 2°, and 0.2 mm, respectively. The test angle varied from 2° to 120°, and the scanning mode was step-scan with a step length and scanning speed of 0.1° and 3 s/step.

The 70 Fourier transform infrared (FTIR) spectroscopy was purchased from BRUKER VERTEX, Germany. The signal-to-noise ratio and sampling rate were 55,000:1 (peak value tested in 1 min) and 80 spectra/s (spectral resolution of 16 cm^−1^). Moreover, the measurement range of spectral area, step-scan time resolution, and resolution were 30,000 to 10 cm^−1^, 5 ns, and 0.4 cm^−1^, respectively.

For the zeta potential analyzer (Colloidal Dynamics Zeta, USA), the colloidal particle size of the zeta potential ranged from 1 nm to 50 μm, and the pH value varied from 0.5 to 13.5. Moreover, the test range of electrical conductivity was 0 to 5 S/m and the resolution of the titration apparatus was 1 μL.

### Experimental methods

By combining the static adsorption method with ultraviolet spectrophotometer test, the distribution of adsorption capacity of the surfactant at different concentrations with time was assessed. The adsorption kinetic characteristics were studied by fitting the pseudo-first- and pseudo-second-order kinetic models. Through the SEM, EDS, and FTIR tests, this study tested the surface morphology and adsorption characteristics of montmorillonite before, and after, adsorption. The adsorption characteristics of the interlayer of montmorillonite after the adsorption were examined on the basis of the XRD test data. Through use of a zeta potential test, the influences of pH value on the adsorption of montmorillonite were analyzed and the specific experimental steps are described as follows:

1. Determination of adsorption capacity and research into adsorption kinetic characteristics
  There are many methods of measuring the adsorption capacity of a liquid on the surface of a solid. The UV spectrophotometry is simple and accurate and, therefore, was used as follows:
  1. Some 8.712 g of SDBS was weighed to a precision of 0.001 g and dissolved in distilled water to prepare a 0.025 mol/L SDBS solution.
  2. By using a volumetric flask, a stock SDBS solution with a concentration of 0.025 mol/L was used to prepare solutions with concentrations of 3 mmol/L, 4 mmol/L, and 5 mmol/L. Conducting wavelength scanning using the ultraviolet spectrophotometer on SDBS solutions with different concentrations, the standard curves were established to determine their SDBS contents.
  3. Five samples were weighed and the mass of each sample was 2.000 g: the montmorillonite was then measured and put into a 250-mL conical flask. The SDBS solutions (200 mL) at different concentrations (3, 4, and 5 mmol/L) were added separately to different conical flasks. Stirring the solution by a magnetic stirrer, the adsorption tests of the solution were carried out at different times (20, 40, 60, 90, and 120 min). In addition, the solution after the adsorption was centrifuged at 10,000 rpm. The supernatant was collected and filtered twice through a 0.22-μm membrane filter, so as to remove the residual montmorillonite particles, therein, and eliminate the effects of montmorillonite particles on the absorbance test, thus reducing experimental error therein. By using the ultraviolet spectrophotometer to measure the absorbance of the filtered solution and comparing it with the standard concentration–absorbance curve, the adsorption capacity could be calculated. The earlier operation was repeated to test the adsorption capacity of 3, 4, and 5 mmol/L SDBS solutions, respectively.
  4. By using the classical pseudo-first- and pseudo-second-order kinetic models for fitting, the adsorption kinetic characteristics were obtained through comparison.
2. Changes in surface and interlayer adsorption of montmorillonite after adsorption
  1. The mixed solution comprising SDBS and montmorillonite after adsorption was centrifuged. The precipitates were filtered and dried in the drying oven. The samples before, and after, adsorption were observed using SEM and analyzed by EDS.
  2. The montmorillonite samples were ground by a mortar and screened to less than 200 mesh size before being placed in a drying oven for 24 h. The dried samples were placed in a clean beaker and were tested by XRD.
  3. Dried montmorillonite sample of 2.000 g before, and after, absorption was separately placed in various beakers. The dried sample was mixed with KBr (1.500 g) to form samples with a concentration ratio of 1:150. The sample was dried in a drying oven for 24 h. Afterward, the sample was compressed and tested using the infrared spectrometer.
3. Influences of pH value on the adsorption capacity of montmorillonite

Some 2.000 g of dried montmorillonite sample was separately placed in different beakers. At a solid–liquid ratio of 1:100, deionised water and SDBS at different concentrations (3, 4, and 5 mmol/L) were added to different beakers, and the mixing solution was stirred at 25°C for 2 h. Afterward, the potential of the treated solution was tested by a zeta potential analyzer (Colloidal Dynamics, USA).

## Experimental results and analysis

### Adsorption kinetic characteristics

**Figure 1:**
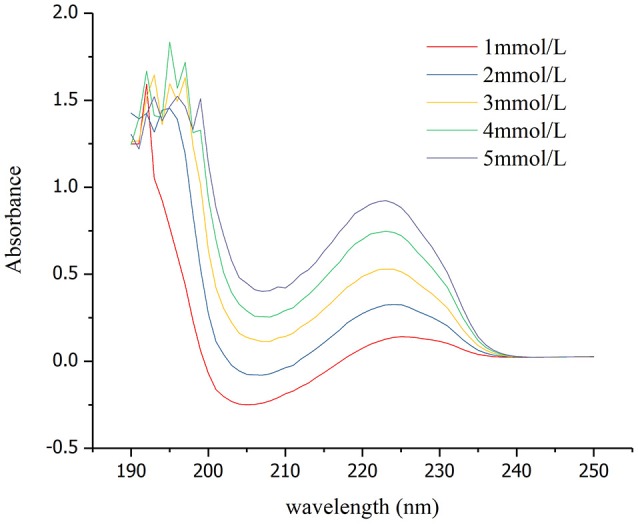
Relationships between absorption wavelength and absorbance.

**Figure 2:**
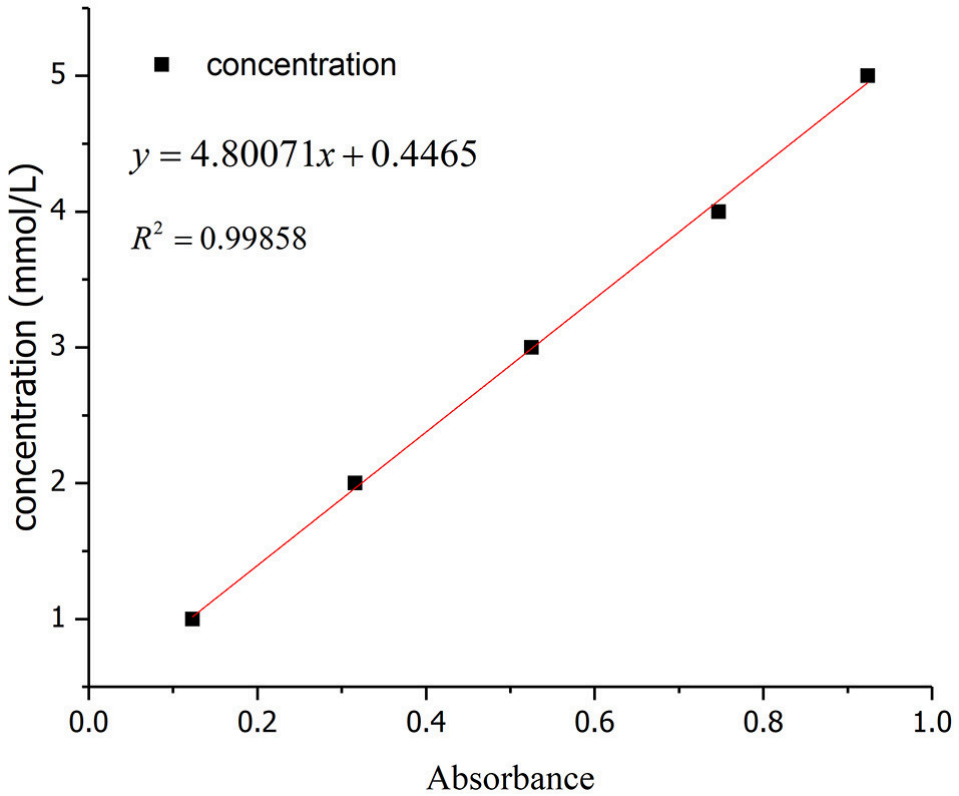
Relationships between concentration and absorbance.

1. Adsorption capacity of the solution with different concentrations
  1. Measurement of the optimum wavelength of absorption
    The absorbance of wave peak for the solution at different concentrations (1, 2, 3, 4, and 5 mmol/L) was measured by ultraviolet spectrophotometer. The peak wavelength of absorbance of SDBS was stable at about 223 nm under these experimental conditions (Figure 1). The linear correlation between the peak absorbance and concentration of SDBS at 3 to 5 mmol/L was preferable, and the correlation coefficient *R*^2^ exceeded 0.99 (Figure 2).
2. The calculated adsorption capacity of solutions of different concentrations
  The absorbance at different concentration (3, 4, and 5 mmol/L) was measured and the adsorption capacity was calculated as follows:
  (1) $$\begin{array}{r} Q=\frac{\left(C_{0}-C\right)V}{m} \end{array}$$

Where, *Q* is the adsorption capacity (mmol/g); *C*_0_ and *C* are initial concentration and the concentration after adsorption, respectively (mmol/L); *V* is volume of the solution (L); and *m* represents the mass of the montmorillonite (g).

**Figure 3:**
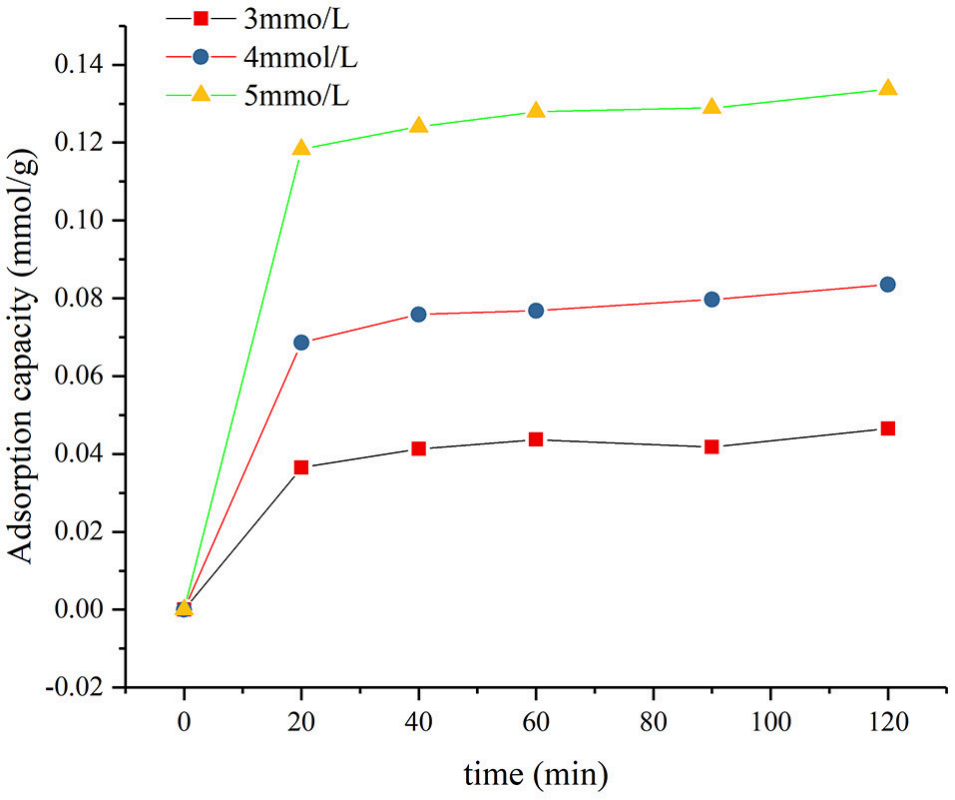
Changes in adsorption capacity of montmorillonite with time.

The adsorption capacity of SDBS solutions with different concentrations on a montmorillonite sample was calculated according to formula (1). The results are illustrated in Figure 3.

In Figure 3, the adsorption capacity of montmorillonite with three different concentrations increased slowly over time. This increase slowed gradually, and the adsorption reached equilibrium after 2 h. The adsorption capacity increased with the concentration of SDBS.

2) Kinetic characteristics of adsorption

To study further the adsorption behavior of SDBS solution on montmorillonite, the typical pseudo-first-order kinetic model of Lagergren and pseudo–second-order kinetic model of McKay were applied.

The pseudo-first-order kinetic equation (Lagergren, 1898) was mainly used to present the adsorption of single molecules in surfactant. The adsorption of surfactant on the solid–liquid interface can be regarded as an equilibrium reversible process between solution phase and solid phase and expressed as:

(2) $$\begin{array}{r} d\theta /dt=k_{\alpha }C_{0}\left(1-\theta \right)-k_{d}\theta \end{array}$$

Where, θ is surface coverage of the surfactant; (1−θ)refers to the surface exposure rate; *k*_α_ and *k*_*d*_ represent the adsorption and desorption equilibrium constants, respectively.

Where, θ can be expressed as:

(3) $$\begin{array}{r} \theta =Q/Q_{m} \end{array}$$

From Equation (3):

(4) $$\begin{array}{r} Q_{t}=\frac{C_{0}k_{\alpha }Q_{m}}{C_{0}k_{\alpha }+k_{d}}\left[1-\exp\left(-\left(C_{0}k_{\alpha }+k_{d}\right)t\right)\right] \end{array}$$

The initial adsorption capacity is small, so *Q*_*m*_ ≈ *Q*_*e*_, and Equation (4) can be expressed as:

(5) $$\begin{array}{r} \frac{Q_{t}}{Q_{e}}=1-\exp\left(-k_{1}t\right) \end{array}$$

Where, *Q*_*e*_ and *Q*_*t*_ are, respectively, the equilibrium adsorption capacity of surface-active agent on the adsorbents and the adsorption capacity at time *t*, mg/g; *k*_1_ (*k* = *C*_0_*k*_*a*_ + *k*_*d*_) is the pseudo-first-order adsorption constant (min^−1^).

Equation (5) can be rewritten as Equation (6):

(6) $$\begin{array}{r} \ln\;\left(Q_{e}-Q_{t}\right)=\ln\;Q_{e}-k_{2}t \end{array}$$

The pseudo-second-order adsorption model (Ho and Mckay, 1999) was established by the equilibrium adsorption capacity and adsorption capacity at different times, and can be described as:

(7) $$\begin{array}{r} \frac{dQ_{t}}{dt}=-k_{2}{\left(Q_{e}-Q_{t}\right)}^{2} \end{array}$$

Equation (7) is calculated based on an integral method, when *t* = 0 to *t* = *t* and *Q*_t_ = 0 to *Q*_t_ = *Q*_e_; it is found that:

(8) $$\begin{array}{r} \frac{t}{Q_{t}}=\frac{1}{k_{2}Q_{e}^{2}}+\frac{1}{Q_{e}}t \end{array}$$

Equation (8) is converted into:

(9) $$\begin{array}{r} Q_{t}=Q_{e}^{2}k_{2}t/\left(1+k_{2}Q_{e}t\right) \end{array}$$

Where, *k*_2_ is an adsorption constant, g.mg^−1^.min^−1^; *Q*_*e*_ represents the equilibrium adsorption capacity of surfactant, mg/g; *Q*_t_ denotes the adsorption capacity of the surfactant on the surface of the solid at time *t*, mg/g; and *k*_2_*Q*_*e*_^2^ is the initial adsorption time, h.

The pseudo-first-order and pseudo-second-order kinetic equations show that adsorption capacity of two models at time *t* has a nonlinear relationship with time; however, owing to the final equation being deduced by mathematical model, when the adsorption kinetics equation is fitted, the physical meaning of all equations must be satisfied. Therefore, based on Equations (5), (6), (8), and (9), the adsorption capacity at different times is fitted by using nonlinear Eqs (5) and (9). According to Equation (2), the adsorption rate constants at different concentrations were calculated. Research shows that fitting result are more likely to produce large errors by using a single testing parameter (R^2^ OR R^2^ adj) and cannot accurately reflect the fitting results. Therefore, 11 kinds of error functions were used to analyze the results of nonlinear fitting, and the adsorption kinetic model of montmorillonite was obtained. (Hadi et al., 2010). The fitting and calculated results are summarized in Table 1.

**Table 1:** Adsorption kinetics and adsorption rate of montmorillonite under different SDBS concentrations.

***C*_SDBS_(*mmol*/*L*)**	**Pseudo-first order kinetic model**		**Q_e_(exp)/mmol/g**	**Q_e_(cal)/mmol/g**	**k/min^−1^**
3	Nonlinearity	Q_t_ = 0.04383 × (1–e^−0.08649 × *t*^)	0.0468	0.0438	0.0864
4		Q_t_ = 0.07965 × (1–e^−0.09556 × *t*^)	0.0842	0.0797	0.0955
5		Q_t_ = 0.12912 × (1–e^−0.12085 × *t*^)	0.1337	0.1291	0.1208
***C*_SDBS_(*mmol*/*L*)**	**Pseudo-second order adsorption model**		**Q**_e_**(exp)/mmol/g**	**Q**_e_**(cal)/mmol/g**	**k/min**^−1^
3	Nonlinearity	Q_t_ = t/(122.9994 + 21.2314 × t)	0.0468	0.0471	3.0478
4		Q_t_ = t/(57.6985 + 11.7744 × t)	0.0842	0.0849	1.8454
5		Q_t_ = t/(21.5147 + 7.4338 × t)	0.1337	0.1345	1.9019

As can be seen from Tables 1, 2, the comparison of Q_e_ (experimental value) and Q_e_ (calculated value) showed that calculated values of the pseudo-second-order adsorption kinetics model are larger than the experimental values, and the calculated values of the pseudo-first-order adsorption kinetic model were smaller than the experimental values. The error function calculation results of the pseudo-second-order adsorption kinetics model were smaller than that of the pseudo-first-order adsorption kinetics model. The calculated values of the pseudo-second-order adsorption kinetics model are closer to the experimental values. The results indicate that the adsorption of SDBS on montmorillonite was consistent with a pseudo-second-order kinetic equation, and its adsorption was mainly via chemisorption.

**Table 2:** Values of the error functions of the adsorption kinetics models.

**Error function model**	**Pseudo-first-order kinetic model**			**Pseudo-second-order adsorption model**		
	**3 mmol/L**	**4 mmol/L**	**5 mmol/L**	**3 mmol/L**	**4 mmol/L**	**5 mmol/L**
RMSE(10^−3^)	1.82	2.58	3.11	1.53	1.38	1.59
χ^2^(10^−4^)	3.04	3.36	3.01	2.12	0.95	0.79
G^2^(10^−4^)	4.50	4.87	3.35	2.38	0.82	1.01
ERRSQ(10^−5^)	1.32	2.66	3.87	0.94	0.76	1.02
HYBRD(10^−4^)	2.99	3.32	3.0	2.18	0.95	0.78
MPSD(10^−3^)	6.79	4.15	2.32	5.07	1.19	0.61
ARE	0.15	0.12	0.08	0.12	0.06	0.04
EABS(10^−3^)	6.48	9.36	10.61	5.03	5.11	6.33
APE%	3.00	2.39	1.66	2.31	1.29	0.98
AIC_C_	−70.14	−65.96	−63.70	−72.19	−73.47	−71.71
Mallows(10^−3^)	7.28	10.31	12.44	6.13	5.51	6.38

As the concentration of the SDBS increased from 3 to 5 mmol/L, the adsorption rate constant *k* (Initial adsorption rate) decreased from 3.0 to 1.9, which indicated that the adsorption rate declined with increasing concentration of the solution, and a high initial concentration of SDBS was disadvantageous to adsorption. The reason is that the higher concentration of SDBS can cause an increase in the repulsive force between the free SDBS molecules in the solution and the molecules adsorbed by montmorillonite, which further caused the decreased initial adsorption rate. In Table 1, the initial adsorption rate of 4 mmol/L was slightly lower than the rate of 5 mmol/L: the reason for this phenomenon may be that monolayer adsorption can quickly form on the surface of Montmorillonite in higher concentration solutions.

### FTIR analysis of montmorillonite before, and after, adsorption

**Figure 4:**
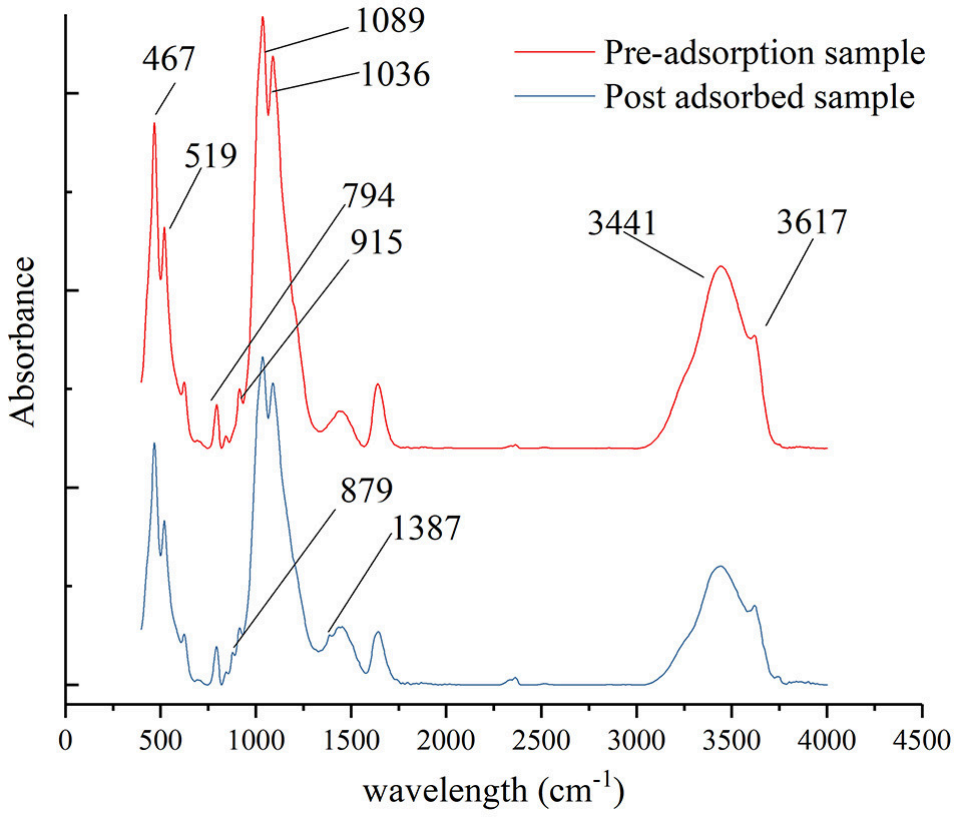
Comparison of infrared spectra of montmorillonite before, and after, adsorption.

The FTIR test was conducted before, and after, the adsorption of montmorillonite. The test results are shown in Figure 4.

As can be seen from Figure 4, the FTIR spectra of montmorillonite samples that adsorbed SDBS were similar to that of the original sample, which indicated that the montmorillonite skeleton did not change before, or after, adsorption. The main features of the original montmorillonite peak included: an OH stretching vibration peak at 3,617 cm^−1^ and the big absorption peak at the region around 3,441 cm^−1^ were found, which were caused by interlayer water and adsorbed water. The absorption peaks at 915 and 794 cm^−1^ showed OH^−^ bending vibration. The absorption peak at 1,036 cm^−1^ represented the Si-O-Si adsorption vibration. The absorption peaks at 1,089 cm^−1^ were strong and obvious, and indicated Si-O vibration. The peaks at 519 and 467 cm^−1^ were, respectively, caused by Si-O-Mg and Si-O-Fe vibrations (Lloyd, 1975; Borchardt, 1977; Caillére et al., 1982; Alabarse et al., 2011).

After adsorption of SDBS on montmorillonite, the absorption peaks at 1,387 and 879.3 cm^−1^, respectively, reflected the C-H deformation vibration peak and the stretching vibration peak of the C-C bond (Ohtani et al., 1996). The C-C bond is unique in the aromatic ring, so the presence of C-C and C-H bonds indicated that SDBS was adsorbed on the surface of the montmorillonite through chemical adsorption. The FTIR results demonstrated that the adsorption of SDBS on montmorillonite was via chemical adsorption on the surface of montmorillonite, which is consistent with the fitted results of the adsorption kinetic models.

### Changes to the surface of the montmorillonite before, and after, adsorption

#### SEM analysis of montmorillonite after SDBS adsorption

**Figure 5:**
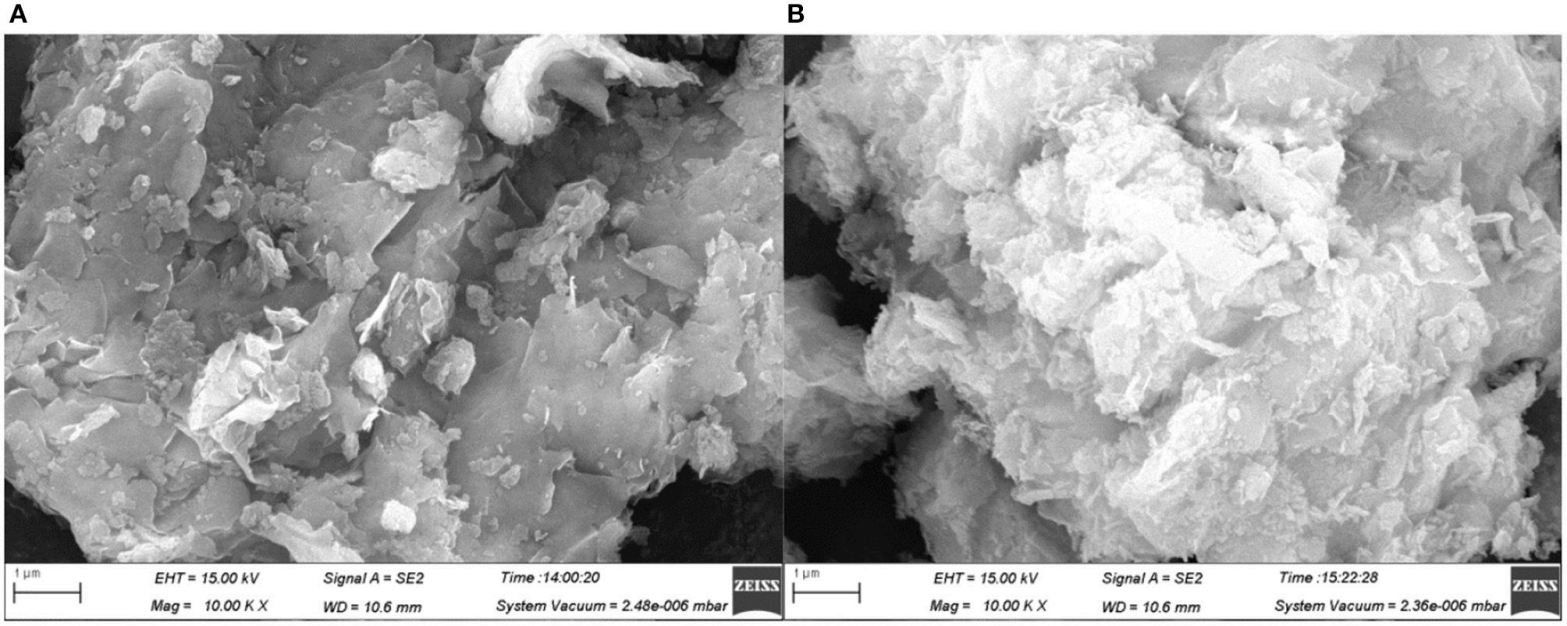
**(A)** The original sample of montmorillonite. **(B)** Sample after 5 mmol/L adsorption.

The SEM observation and EDS analysis can only allow magnified observation and energy spectrum analysis of the local area of particle surface in the samples before, and after, absorption; however, they cannot reveal the overall surface of particles in the samples before, and after, adsorption. Therefore, when observing the surface changes of montmorillonite before, and after, adsorption, this study selected locations where particles showed as similar shapes and dimensions (as far as possible) before, and after, adsorption, so as to measure changes in surface characteristics. The SEM scanning was carried out on the samples before, and after, adsorption and partial results are shown in Figure 5.

As can be seen from Figure 5, the surface of montmorillonite was smooth and had a lamellar structure before adsorption. After adsorption of SDBS on surface, the surface of the montmorillonite became rougher, floc appeared, and the number of pores increased. This indicated that after SDBS was adsorbed onto the surface of montmorillonite minerals, it might change the surface morphology thereof. Owing to the surface morphology of the samples having been observed under the SEM, to study the changes in adsorption characteristics of montmorillonite surfaces, EDS supplementary tests in SEM observation areas were conducted.

#### EDS analysis of montmorillonite after adsorbing SDBS

**Figure 6:**
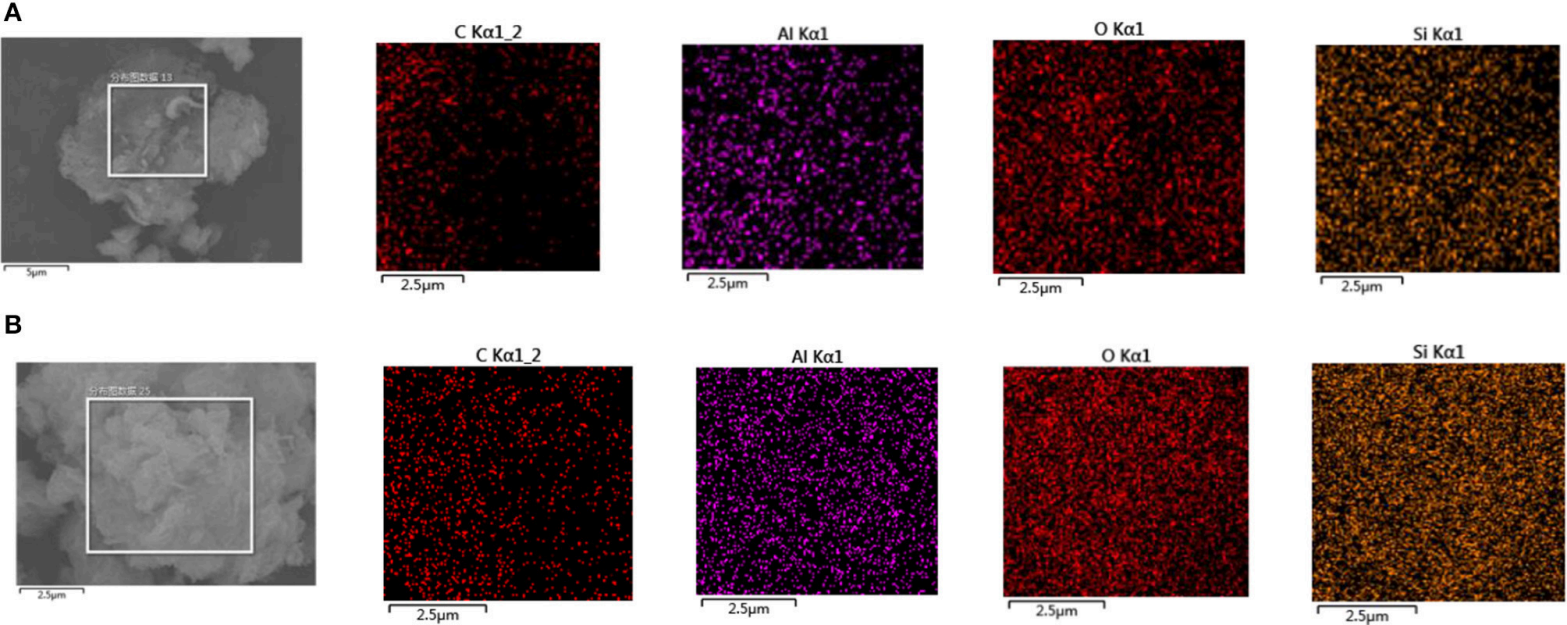
**(A)** EDS scanning diagram of the original montmorillonite sample. **(B)** EDS scanning diagram of montmorillonite samples after adsorption of SDBS.

The surface of montmorillonite samples before, and after, adsorbing SDBS was scanned by EDS, and partial results are shown in Figure 6.

As can be seen from Figure 6A, the surface of original samples was mainly composed of Al, O, and Si components with the contents, in a decreasing order, being O, Si, then Al. The distribution of the three components was uniform. The content of elemental Si was higher than that of Al. After the adsorption of SDBS on montmorillonite, elemental C appeared on the surface of the samples. As can be seen from Figure 5B, the punctate distribution of C was similar to the distribution of rough surface areas in the tested montmorillonite sample; this indicates that the surface roughness of montmorillonite may be due to the adsorption of SDBS. The distribution of O, Al, and Si elements was uniform, and the distribution density of the elements, in a decreasing order, was O, Si, then Al.

The samples, after the adsorption of different concentrations of SDBS (3, 4, and 5 mmol/L), were scanned by EDS. The main atomic content changes are displayed in Table 2.

As can be seen from Table 3, the main components of montmorillonite were SiO_2_ and Al_2_O_3_, and in some aluminum octahedral layers, Al^3+^ was replaced by Mg^2+^. The C elements in the original samples mainly came from carbonate compounds. With the increase in concentration, the percentage content of C atoms on the surface increased from 14.05 to 28.56%. Compared with the increment in the C/Si ratio, the O/Si ratio showed a smaller increase from 1.918 to 2.330. This is because O, and a small amount of C, were contained in the structure of the montmorillonite, while SDBS had a high C content. This further verified that the higher the concentration, the greater the adsorption capacity on the montmorillonite surface.

**Table 3:** The main atomic content changes on the surface of montmorillonite after SDBS adsorption.

**Samples**	**C%**	**O%**	**Na%**	**Mg%**	**Al%**	**Si%**	**O/Si**
Original	14.05	50.01	1.56	1.99	7.11	25.28	1.981
After 3 mmol/L adsorption	18.66	44.2	1.48	0.84	4.76	20.95	2.109
After 4 mmol/L adsorption	22.47	45.82	1.44	1.57	4.89	21.73	2.108
After 5 mmol/L adsorption	28.56	44.74	1.2	1.59	4.71	19.2	2.330

### Changes in the interlayer structure before, and after, the adsorption of SDBS on montmorillonite

**Figure 7:**
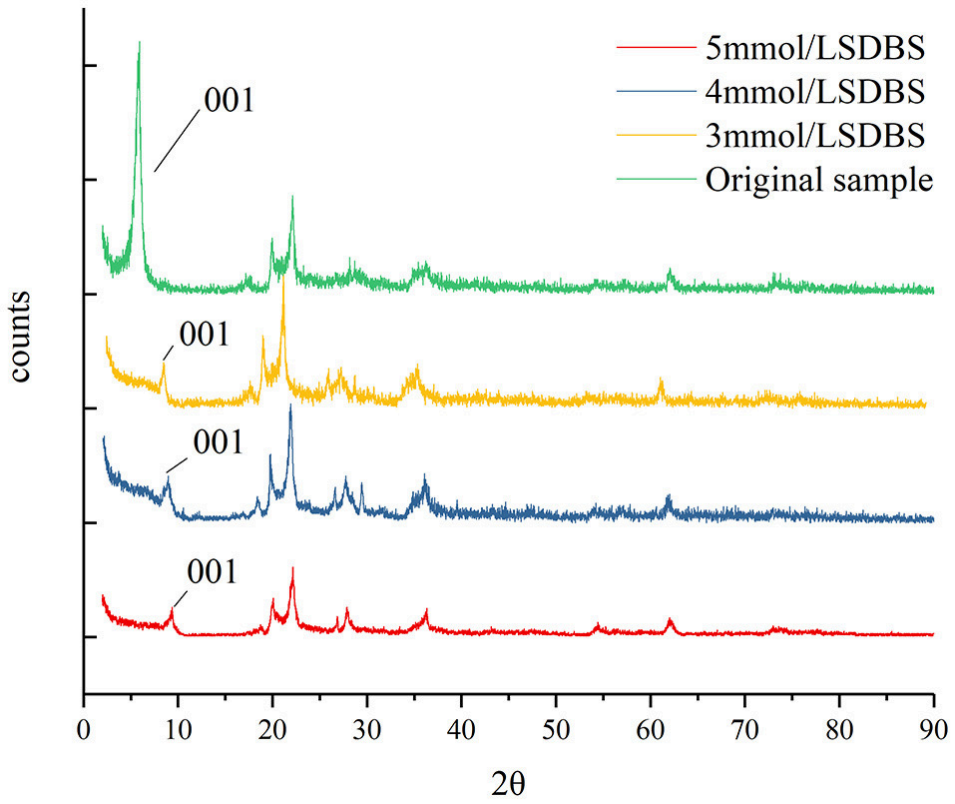
Comparison program of XRD testing results before, and after, adsorption.

The XRD test was carried out before, and after, the adsorption of montmorillonite. The test results are shown in Figure 7.

As can be seen from Figure 7, after the montmorillonite samples adsorbed SDBS, the 001 characteristic peaks were shifted, and the peak of the *d*_001_ peak decreased, which showed that the structure of montmorillonite changed after adsorbing SDBS. The interlayer spacings, before and after montmorillonite adsorption, were calculated using the Prague equation: *d*_001_ of the original sample was 1.4970 nm (Rosenquist, 1959; Bergaya et al., 2006), and *d*_001_ was 1.0371 nm after absorbing SDBS; as the concentration of SDBS solution increased from 3 to 5 mmol/L, *d*_001_ decreased to 0.9437 nm. The changes in interlayer spacing indicated that SDBS was adsorbed on the interlayer of the montmorillonite, and with increasing SDBS concentration, the adsorption capacity of SDBS was enhanced in the interlayer of the montmorillonite, and the number of hydrophobic groups increased so that the interlayer water content decreased, resulting in the decreased interlayer spacing.

The absorption peak of different SDBS concentrations before, and after, adsorption can be seen: as the concentration increased, the amplitudes of the peak were reduced. The adsorption of SDBS on the surface of the montmorillonite may be affected and the XRD testing results may reflect the adsorption of SDBS on the surface of the montmorillonite.

It was concluded that the surface and layer of montmorillonite both adsorbed SDBS, and the participation of interlayer adsorption increased the adsorption capacity of montmorillonite to SDBS.

### The effect of pH on the adsorption of montmorillonite

**Figure 8:**
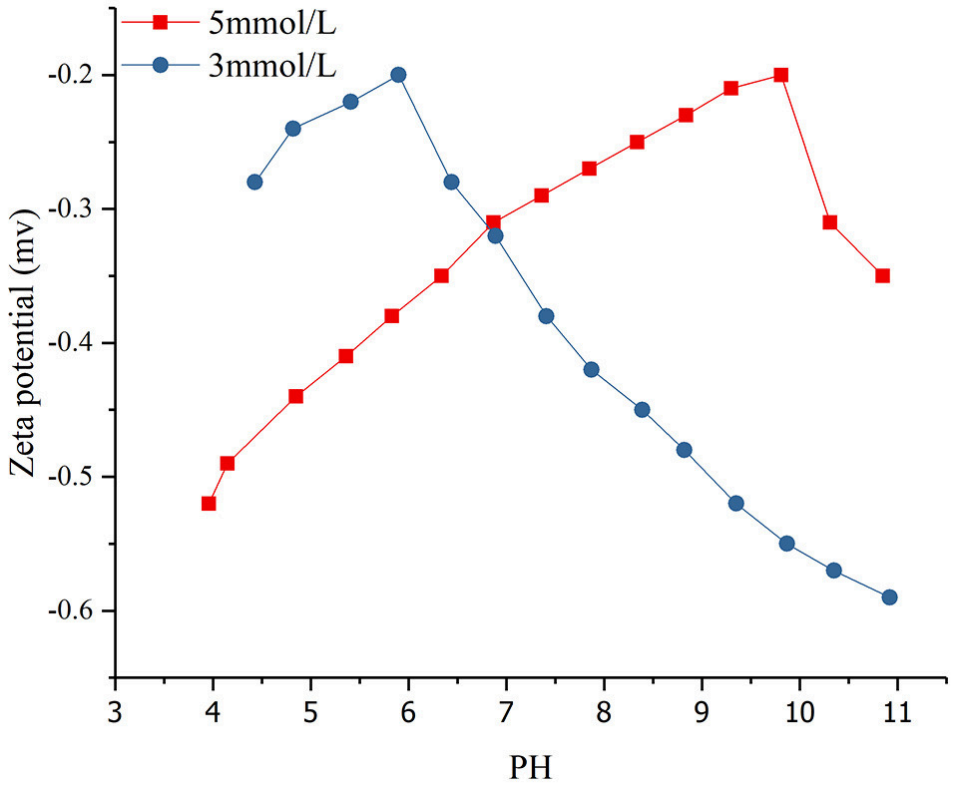
Zeta potential distribution of montmorillonite surface at different pH values in various SDBS concentrations.

Zeta potential testing was conducted on the solution with different concentrations of SDBS (3 and 5 mmol/L) after adsorption on montmorillonite (Figure 8).

Research has shown that the zeta potential of montmorillonite in distilled water was between −25 mV and −37 mV and only shows slightly decline as the pH increases from 2.80 to 10.10 (Saka and Güler, 2006; Navrátilová and Maršálek, 2012; Yu et al., 2018). Compared with previous studies, the absolute value of the montmorillonite zeta potential in the SDBS solution was significantly lower than that in the distilled water. The smaller the absolute value of Zeta potential, the worse the suspension of montmorillonite and the greater the settlement. The reason for this was that the SDBS adsorbed by montmorillonite under the effect of hydrophobic force reduced the montmorillonite suspension and increased the amount of sedimentation.

The concentration of SDBS (Figure 8) was 3 and 5 mmol/L, and the initial pH for the mixed solution of montmorillonite and SDBS was about 10.9. The range of pH values was 4 to 11 and the zeta potential was negative. When an HCl solution was added to the mixed solution, the OH^−^ content of the solution was neutralized with H^+^, and the electrostatic repulsion force of montmorillonite surface was decreased. Also, the hydrophobic force did not change. As the suspension of montmorillonite decreased and the sedimentation, the adsorption capacity of SDBS on the surface of montmorillonite increased. As the concentration of H+ continued to increase, the electrostatic repulsion force on the surface of the montmorillonite decreased, and a more obvious agglomeration and greater decrease in zeta potential were found. Also, the adsorption capacity of SDBS on the surface of montmorillonite continued to increase. When the absolute value of the zeta potential was the smallest, the amount of precipitation was the largest, and the adsorption capacity of the surface reached a maximum. As the concentration of H^+^ continued to increase, the redundant H^+^ in the solution reacted with SDBS radicals to form SDBS acid, and the absolute pH value of the solution was increased.

As the concentration of SDBS in the solution was increased from 3 to 5 mmol/L, the hydrophobic force of SDBS was increased. For cases at the same pH, the higher the concentration of SDBS, the more obvious the aggregation, and smaller absolute value of zeta, the greater adsorption capacity of SDBS on the montmorillonite surface. With increasing H^+^ concentration, the absolute value of the zeta potential was apt to decrease to a minimum at higher concentrations, and the adsorption capacity of SDBS on the surface of the montmorillonite was weakened, quickly reaching a maximum. The increasing concentration made the surface of the montmorillonite form a double-layer adsorption pattern. When the adsorption capacity reached its maximum, as the concentration of H^+^ increased, the greater was the concentration of SDBS and more SDBS acid reacted with H^+^, which caused a greater zeta potential to be measured in the solution.

It was concluded that with the addition of H^+^, the negative charge on the montmorillonite surface was reduced, and the absolute value of the zeta potential of montmorillonite decreased, the agglomeration effect was more obvious, and the amount of SDBS adsorbed on the montmorillonite surface increased. This further verified that the electrostatic repulsion on the montmorillonite surface would hinder the adsorption of SDBS.

## Conclusions

1. The adsorption capacity of SDBS at different concentrations on montmorillonite was gained by spectrophotometric tests. The adsorption was more consistent with that of adsorption simulated by the pseudo-second-order kinetic model. The higher the concentration, the greater the adsorption capacity of SDBS on montmorillonite. The montmorillonite adsorption to SDBS is chemical adsorption. The FTIR test has further verified this conclusion.
2. The morphology and interlayer structure in the surfactant-adsorbing sample montmorillonite in the solution containing different concentrations of SDBS and the nonadsorbed sample were tested by using SEM–EDS and XRD, respectively. The results showed that the surface of montmorillonite in a surfactant-adsorbing solution became coarser, and the surface properties of the sample changed. In EDS testing, as the concentration of SDBS in solution increased, the amount of SDBS adsorbed on the surface of the montmorillonite increased. The XRD results suggested that in SDBS solution, the higher the concentration of SDBS, the greater the amount of SDBS adsorbed on the surface of the sample interlayer. As the SDBS hydrophobic groups adsorbed on the interlayer of sample montmorillonite increased in number, the amount of interlayer water decreased, and the interlayer spacing decreased. In conclusion, interlayer adsorption and surface adsorption coexisted in the process of adsorption of SDBS by the montmorillonite.
3. Based on the results of zeta potential tests, it can be seen that montmorillonite adsorbed anionic surfactant, and electrostatic repulsion on the surface hindered the adsorption. The addition of H^+^ reduced electrostatic repulsion and increased the adsorption capacity.
4. There are many factors influencing the adsorption of SDBS on montmorillonite, such as temperature, concentration, and electrolyte. Due to the limitations of experimental conditions and time, this research only investigated the adsorption characteristics of SDBS on the surface of montmorillonite at room temperature (25°C) and at different pH values. Moreover, in the research on adsorption kinetic characteristics, only kinetics equations were used for fitting. In the future, it is necessary to study other influencing factors and adsorption kinetic characteristics from the perspective of molecular simulation, so as to reveal the overall adsorption mechanisms acting therein.

## Author contributions

XN was mainly responsible for the editing and writing of the article. The experiment in the article was carried out by ZL and YW.

### Conflict of interest statement

The authors declare that the research was conducted in the absence of any commercial or financial relationships that could be construed as a potential conflict of interest.
